# Retraction statement

**DOI:** 10.1080/10495398.2026.2689211

**Published:** 2026-06-25

We, the Editor and Publisher of Animal Biotechnology, have retracted the following article:

Chang, C., He, X., Di, R., Wang, X., Han, M., Liang, C., & Chu, M. (2024). Thyroid transcriptomic profiling reveals the differential regulation of lncRNA and mRNA related to prolificacy in Small Tail Han sheep with *FecB^++^* genotype. *Animal Biotechnology*, *35*(1). https://doi.org/10.1080/10495398.2023.2254568

Following publication, concerns have been raised about the integrity of the data presented in the article. When approached for an explanation, the authors responded, however, they were unable to address the concerns raised. As verifying the validity of published work is core to the integrity of the scholarly record, we are therefore, retracting the article. The authors do not agree with the retraction.

We have been informed in our decision-making by our editorial policies and the COPE guidelines. The retracted articles will remain online to maintain the scholarly record, but they will be digitally watermarked on each page as ‘Retracted’.

